# Composites of Natural-Polymer-Cross-Linked Poly(ortho-phenylenediamine)-Grafted SiO_2_ for Removal of Anionic and Cationic Dyes from Wastewater

**DOI:** 10.3390/polym17212818

**Published:** 2025-10-22

**Authors:** Sara A. Alqarni

**Affiliations:** Department of Chemistry, College of Science, University of Jeddah, Jeddah, Saudi Arabia; sasalqarni@uj.edu.sa or sara.science.college@gmail.com

**Keywords:** SiO_2_ nanoparticles, chitosan, chitin, guar gum, poly (ortho-phenylenediamine), dye removal, kinetics, isotherm

## Abstract

This study synthesizes three new composites: chitin-cross-linked poly(ortho-phenylenediamine)-grafted silicon dioxide (CT-PoPD-grafted SiO_2_), chitosan-cross-linked PoPD-grafted SiO_2_ (CS-PoP-grafted SiO_2_), and guar-gum-cross-linked PoPD-grafted SiO_2_ (GG-PoPD-grafted SiO_2_). These biopolymer-based materials were developed as cost-effective, biocompatible adsorbents with increased surface area for removing Acid Red 1 AR1) and Crystal Violet (CV) dyes. Structural and morphological analyses through Fourier-transform infrared spectroscopy, X-ray diffraction, and X-ray photoelectron spectroscopy (XPS) confirmed their successful synthesis. Adsorption studies were conducted under various conditions, including adsorbent dosage, pH, temperature, and contact time. Among the composites, GG-PoPD-grafted SiO_2_ demonstrated superior performance, achieving 99.1% and 95.6% removal of AR1 and CV, respectively. Kinetic analysis revealed a pseudo-second-order model, while thermodynamic results indicated a spontaneous and endothermic adsorption process. In conclusion, the GG-PoPD-grafted SiO_2_ composite exhibits significant potential as an effective and sustainable material for wastewater treatment.

## 1. Introduction

Industrial effluents from various sectors, including the food, paint, textile, printing, and pharmaceutical industries, significantly contribute to global water pollution. Hazardous pollutants in industrial waste streams have led to numerous health and environmental challenges. The ability of inorganic contaminants to accumulate in living organisms is associated with their high concentrations in groundwater [1]. Approximately 10–15% of harmful dye pollution originates from the textile industry [2]. Dyes can be categorized based on properties such as azo, basic, acidic, dispersion, reactive, and metal complex. The detrimental characteristics of industrial effluents containing dyes pose a major concern, disrupting ecological balance and endangering aquatic life, humans, and plant species [3,4,5]. The primary focus of this study was on the AR1 and CV model dyes. AR1 dyes are commonly used for coloring wool, leather, and silk, characterized by aromatic rings and azo linkages. These complex chemical compounds exhibit mutagenic and antibacterial properties, posing hazards to aquatic creatures and humans [6]. In contrast, CV dye is known to have adverse effects on human health. It can cause ocular burns, potentially leading to irreversible corneal or ocular damage. Inhalation exposure may trigger a brief episode characterized by rapid or labored breathing, nausea, vomiting, excessive sweating, hypermotility, diarrhea, and abdominal pain [7]. Various methods are available to address wastewater contamination, including electrocoagulation, chlorination, coagulation, flotation, filtration, ozonation, membrane separation, chemical oxidation, adsorption, and ultrafiltration. Traditional water treatment methods are energy-intensive and rely on non-sustainable synthetic materials [8,9]. Adsorption studies focus on developing innovative adsorbents with high surface areas, substantial adsorption capacity, and cost-effectiveness. Materials like fly ash, clay, and zeolite have been successfully used for environmental remediation in a cost-effective manner [10]. In contrast, although certain adsorbents effectively remove dyes, many existing materials exhibited a limited number of active sites, reduced surface areas, and insufficient kinetic adsorption characteristics. Recently, nanoadsorbents have gained attention as promising materials for advancing adsorption technologies [11]. These nanoadsorbents represent a new class of adsorbents that have been effectively utilized for the removal of various pollutants from aquatic environments. SiO_2_ nanoparticles [12], TiO_2_ nanoparticles [13], CuO nanoparticles [14], MWCNTs, ZnO [15], AgTiO_2_ [16], and their composites have been used to eliminate various organic compounds, organic dyes, and heavy metals [17] from water. Adsorption using biomaterials like chitin (CT), chitosan (CS), and guar gum (GG) has shown promise as a clean and effective alternative method. These natural polymer adsorbents offer functional groups, including hydrophilic and hydrophobic carboxyl groups and amino acids (e.g., –C=O, –OH, and–NH_2_), which serve as effective adsorption sites for a wide variety of aqueous contaminants, thereby enhancing their performance [18]. CS possesses several advantageous properties, including biocompatibility, biodegradability, hydrophilicity, low cytotoxicity, and antibacterial activity, making it highly suitable for water extraction and purification [19,20]. Its abundance and strong adsorption capacity further enhance its practical value. Owing to the presence of hydroxyl and amino groups, CS serves as an effective adsorbent for both anionic and cationic dyes by providing active sites for adsorption [21]. CT, the second most prevalent natural polymer after cellulose, exhibits biodegradability, biocompatibility, and nontoxicity [22]. Additionally, CT nanofibers, with abundant functional groups, such as carboxylate and amino groups, demonstrate effective water dispersion and present potential as biological adsorbents for pollutant removal in aqueous environments [23]. GG is a biopolymer known for its multiple hydrophilic functional groups, making it an effective adsorbent [24]. Moreover, GG is a biodegradable, non-toxic, and naturally abundant material. It is biocompatible, does not accumulate in biological systems, and exhibits significant swelling behavior, indicating favorable hydrogel properties in its unmodified state. However, certain limitations restrict the widespread application of GG for this purpose. Modification of its biopolymer backbone has proven effective in enhancing activity and enabling the design of materials tailored to specific requirements [25]. Literature reports indicate extensive research on the chemical modification of GG through grafting techniques, including radiation initiation, enzymatic processes, plasma-induced grafting, free radical initiation, ring-opening polymerization, and click chemistry [26]. Various GG-based materials, such as zeolite imidazolate framework (ZIF-8)-GG–polyvinylpyrrolidone [27], carboxymethyl-hydroxypropyl GG [28], and GG-grafted poly(hydroxyethyl methacrylate) [29], have been employed as effective dye adsorbents. Ortho-phenylenediamine (oPD), a conductive polymer, has garnered attention for its potential in water pollutant removal due to its unique electrical properties, low-cost monomers, ease of synthesis, resistance to environmental stresses, and reversible properties. The adsorption efficiency of oPD is further enhanced by functional groups such as NH-. Nanocomposites based on oPD exhibit large surface areas, excellent dispersibility, and synergistic effects, making them well-suited for detailed studies on pollutant adsorption. Consequently, oPD-based materials have been investigated extensively to address various environmental challenges [30,31]. In this context, the primary objective of the present study is to synthesize and characterize novel CT-PoPD-grafted SiO_2_, CS-PoPD-grafted SiO_2_, and GG-PoPD-grafted SiO_2_ composites, and to systematically evaluate their efficiency in removing both anionic (AR1) and cationic (CV) dyes from aqueous solutions and real water samples. The significance of this work lies in demonstrating that integrating natural polymers with conductive polymers and SiO_2_ nanoparticles can produce composites with enhanced adsorption performance, increased active sites, and improved stability. Unlike previous studies that focused on single adsorbents or limited experimental conditions, this study provides a comparative evaluation of three distinct biopolymer-based composites, offering new insights into the design of sustainable and highly efficient dye adsorbents.

## 2. Experimental Analysis

### 2.1. Reagents and Materials

From Sigma Aldrich (St. Louis, MO, USA), chitosan, chitin, guar gum, ethanol, hydrochloric acid (36% HCl, purity ≥99%), phosphoric acid (H_3_PO_4_, 85% *w*/*w*, purity ≥99%), hydrogen peroxide (H_2_O_2_, 30% aqueous solution, purity ≥99%), sulfuric acid (98% H_2_SO_4_, purity ≥98%), polyvinyl pyrrolidone (purity ≥99%), tetraethylorthosilicate (TEOS, purity ≥98%), phenol (purity ≥99%), AR1 dye (dye content ≥98%), and CV dye (dye content ≥98%) were acquired. Reagent-grade oPD (purity ≥99%), ammonium peroxydisulfate (APS, purity ≥98%), pellets of sodium hydroxide (NaOH, purity ≥98%), potassium permanganate (KMnO_4_, purity 99%), and glacial acetic acid (purity ≥99.5%) were obtained from Fisher Scientific (Waltham, MA, USA). All chemicals were of analytical reagent grade (AR grade) unless otherwise specified and were used without additional purification. Deionized water (DW) was used in all experiments. By dissolving 0.05 g of each dye in 100 mL of deionized water, a 500 mg/L stock solution of AR1 and CV dyes was prepared. The stock solution was diluted with DW, and further diluted standard solutions of dyes in the range 5–50 mg/L were prepared. For aquatic adsorption, this technique utilized a Britton–Robinson buffer solution with a pH range from 2 to 10 in conjunction with a NaOH/HCl mixture within the same range.

### 2.2. Apparatus

**Figure 1:**
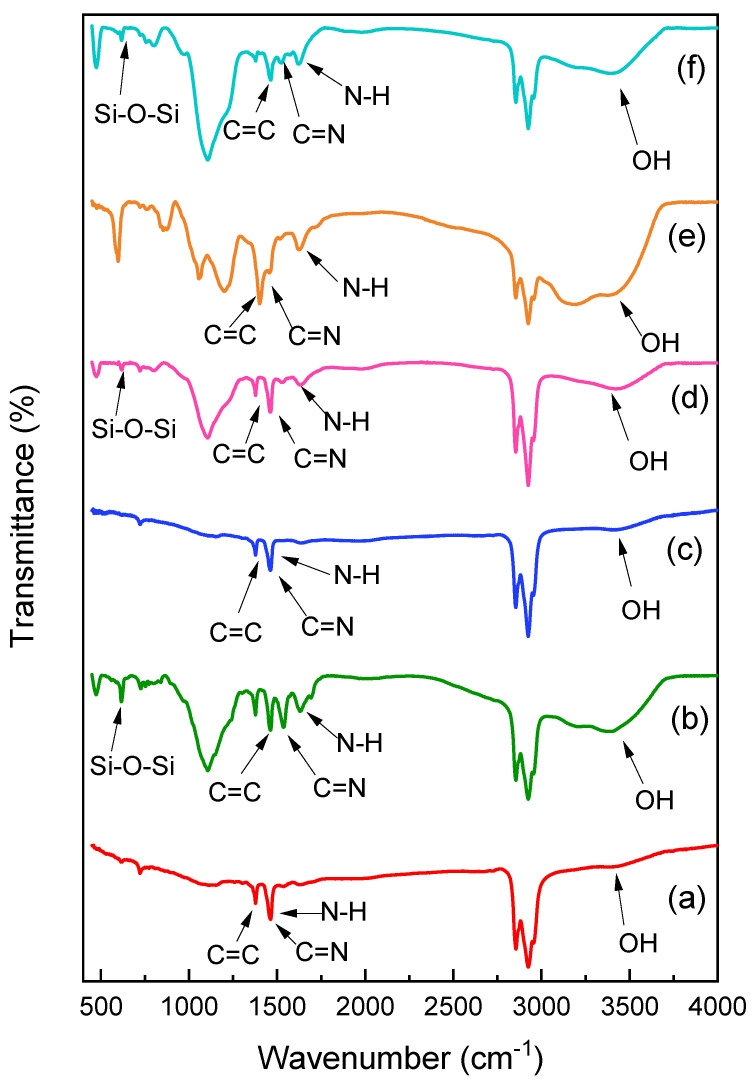
FTIR spectra of CT-cross-linked PoPD composite (**a**), CT-PoPD-grafted SiO_2_ composite (**b**), CS-cross-linked PoPD composite (**c**), CS-PoPD-grafted SiO_2_ composite (**d**), GG-cross-linked PoPD composite (**e**), and GG-PoPD-grafted SiO_2_ composite (**f**).

The micromorphology was examined using scanning electron microscopy (SEM, Quanta 250 FEG Thermo Fisher Scientific, Waltham, MA, USA) with a 30 kV accelerating voltage, 14× magnification, and a maximum resolution of 1,000,000 (Gun.1n). The synthesized materials were analyzed using Fourier-transform infrared (FTIR) spectroscopy with a JASCO spectrometer (JASCO Corporation, Tokyo, Japan). FTIR spectra (Figure 1) were recorded in the wavenumber range of 4000–400 cm^−1^ with a resolution of 4 cm^−1^. Powdered composite samples were finely ground, mixed with spectroscopic-grade KBr, and pressed into pellets prior to measurement, and each spectrum was obtained by averaging 32 scans to improve the signal-to-noise ratio; characteristic peaks were then assigned to the functional groups of the CT-cross-linked PoPD composite, as discussed in the Results section. X-ray photoelectron spectroscopy (XPS) was performed on a Thermo Scientific Escalab 250Xi (Thermo Fisher Scientific, Waltham, MA, USA) using a monochromatic Al-Kα X-ray source. Structural and crystallographic details were obtained via X-ray diffraction (XRD) using a Bruker D8 diffractometer (Bruker AXS GmbH, Karlsruhe, Germany). Additional techniques used in this study included texture analysis, small-angle X-ray scattering, in-plane grazing incidence diffraction, reflectometry, residual stress analysis, and high-resolution diffraction. The absorbance and wavelength of light were measured using a Perkin-Elmer Lambda 25 spectrophotometer (PerkinElmer Inc., Waltham, MA, USA) with a quartz cell of 10 mm path length.

### 2.3. Synthesis of CT-, CS-, and GG-Cross-Linked PoPD Composites

The CT-PoPD composite was synthesized by preparing two separate solutions of CT and oPD [32]. Two grams of CT were mixed with 100 mL of a 4% *v/v* acetic acid solution in a beaker and stirred for 1 h. In another beaker, one gram of oPD was combined with 100 mL of a 0.5 M HCl solution and stirred with a magnetic stirrer for 30 min. The two solutions were then combined in a single beaker and stirred for two hours. Subsequently, 5 g of APS were slowly added to the mixture to facilitate the polymerization of oPD. The grafting process was performed at 0–4 °C for 24 h. The polymerization and grafting solution was neutralized with a NaOH solution. The CT-PoPD solution underwent precipitation with ethanol, followed by filtration with deionized water to remove impurities and oligomers, resulting in a colorless solution. The CT-PoPD suspension was dried in an oven for 24 h at 80 °C. The experiment was repeated twice, replacing CT with CS and GG to produce a CS-cross-linked PoPD composite and a GG-cross-linked PoPD composite, respectively.

### 2.4. Synthesis of CT-, CS-, and GG-PoPD-Grafted SiO_2_ Composites

SiO_2_ was synthesized using the sol–gel method [33]. The synthesis procedure for the CT-PoPD-grafted SiO_2_ composite is as follows: One gram of SiO_2_ powder was sonicated in 100 mL of DW for 2 h. Subsequently, 100 mL of CT-PoPD solution, dissolved in 4% *v/v* AcOH, was added. The reaction mixture was stirred for 24 h under constant agitation at room temperature. A greenish-black precipitate was obtained, followed by filtration with deionized water until the solution became colorless. The final suspension was dried at 80 °C in an oven for 24 h to yield the CT-PoPD-grafted SiO_2_ composite. The CS- and GG-PoPD-grafted SiO_2_ composites were synthesized using the same procedure.

### 2.5. Adsorption Experiment 

We conducted an evaluation to assess the extraction and removal efficiency of (AR1) and (CV) dyes using the quaternary GG-PoPD-grafted SiO_2_ composite solid phase (SP). A 25 mL water solution containing 20 mg/L of AR1 dye was placed in a conical flask, with hydrochloric acid at 0.1 M adjusted to pH = 2, and a specific amount of GG-PoPD-grafted SiO_2_ composite SP (0.015 ± 0.0003 g). In another conical flask, precisely 0.015 ± 0.0003 g of GG-PoPD-grafted SiO_2_ composite SP was added to a 25 mL aqueous solution of CV dye (5 mg/L) at pH = 8. The mixtures were vigorously stirred for two hours. After the aqueous solutions reached equilibrium separation, the remaining dye amounts were measured using a UV-Vis spectrophotometer. Absorbance measurements of the dye in the water solution were taken at 530 nm for AR1 and 590 nm for CV, respectively [34]. both before and after adsorption. The separation efficiency (%E) is calculated using Equation (1), while Equation (2) is utilized to determine the amount of dye adsorbed (q_t_) on the SP.

(1) $$\%\;E=\frac{C_{o}-C_{t}}{Co}\times 100$$

(2) $$q_{t}=\frac{(C_{o}-C_{t})\;V}{m}$$

The initial concentration of dyes is indicated by C_o_, while the remaining concentration of dyes in the solution after agitation is denoted as C_t_.

### 2.6. Applications Study

In order to assess the dye removal and adsorption capabilities of GG-PoPD-grafted SiO_2_ composite SP, water samples were collected from three distinct locations in Saudi Arabia: (a) the Red Sea, (b) wastewater treatment facilities, and (c) laboratory tap water. The samples were filtered through a 0.45 μm membrane, transferred to Teflon containers, and stored at 5 °C in a dark environment. Subsequently, 25 mL of AR1 dye and 25 mL of CV dye were adjusted to pH 2 and 8, respectively, using 0.1 mol/L of HCl. The samples were then shaken with GG-PoPD-grafted SiO_2_ composite SP, and the residual dye concentrations were analyzed spectroscopically. Following the initial measurements, the GG-PoPD-grafted SiO_2_ composite SP used in the study was retrieved, washed with acetone to remove adsorbed dyes, dried, and reused for subsequent dye removal processes.

## 3. Results and Discussions

### 3.1. Characterization of Samples

#### 3.1.1. FT-IR Studies

The analysis of the synthesized CT-cross-linked PoPD composite (a), CT-PoPD-grafted SiO_2_ composite (b), CS-cross-linked PoPD composite (c), CS-PoPD-grafted SiO_2_ composite (d), GG-cross-linked PoPD composite (e), and GG-PoPD-grafted SiO_2_ composite (f) using FT-IR spectroscopy is presented in Figure 1. A prominent peak at approximately 3393 cm^−1^ is attributed to the stretching vibration of the N-H bond in the secondary amine group of the PoPD chain. The stretching vibrations of C=N and C=C in quinoid and benzenoid moieties, respectively, give rise to the bands observed at 1617 and 1524 cm^−1^. The minor band at around 1366 cm^−1^ may indicate the stretching vibration of the C–N imine group. The out-of-plane bending vibration of the benzene ring is detected at 801 cm^−1^ [35]. The symmetric stretching vibrations of the Si-O-Si bond (silanol) are manifested in the bands at 767 and 775 cm^−1^, as shown in Figure 1b,d,f [36]. Additionally, the tetrahedral asymmetric vibrational stretching of Si–O–Si in the SiO_2_ structure is indicated by peaks at 1117 and 1089 cm^−1^ [37]. Both versions of Figure 1 display the FT-IR spectra of CT. The IR spectra of all samples closely resembled the CT spectra [38]. The broad band at 3440 cm^−1^ is assigned to O–H stretching, while N–H stretching is obscured above 3000 cm^−1^ due to hydrogen bonding and overlap with C–H absorptions; however, the presence of N–H is indicated by its bending vibration and the characteristic C=N band. The amide band I, responsible for the peaks at 1654 and 1625 cm^−1^, signifies the presence of the α-CT structure [39]. The amide II band is detected at 1562 cm^−1^. The C–H stretch of methyl CH_3_ groups is indicated by a distinct peak at 1378 to 1314 cm^−1^, and the acetyl group’s Amide III is responsible for the peak at 1260 cm^−1^. The absorption band observed at 1152–1156 cm^−1^ suggests glycosidic connections and C-H stretching vibrations. The distinctive peaks at 1018–1070 cm^−1^ are associated with the saccharide arrangement of the carbohydrate backbone. The glycopyranose bond in CT is confirmed by the signal at 898 cm^−1^ [40]. While there were no shifts in peak positions, the peak intensities of the CT-PoPD-grafted SiO_2_ composite (b) were slightly higher than those of the CT-cross-linked PoPD composite (a). The basic chemical structure of CT remained unchanged, even with the inclusion of SiO_2_ nanoparticles. The FT-IR spectrum of CS (c and d) shows the stretching vibrations of N–H and O–H in secondary alcohols at 3474 cm^−1^, while the peak at 1062 cm^−1^ is associated with these vibrations [41]. In contrast to the bending mode of primary and protonated amines, the absorption band at 1643 cm^−1^ represents the carbonyl stretching mode of amide groups [42]. Various peaks are observed in the FTIR spectra of GG, including: 3427 cm^−1^ for O–H stretching of sugar units, 2889 cm^−1^ for C–H stretching vibrations, 1646 cm^−1^ for ring stretching, and 1177–796 cm^−1^ for the carbohydrate fingerprint [43]. In Figure 1(e and f), the distinctive GG peaks at 1423 and 1008 cm^−1^ correspond to the C–H and O–H bending vibrations, respectively [44]. The effective grafting of GG-PoPD onto SiO_2_ is confirmed by the emergence of new peaks and the reduced intensity of existing peaks in the GG-PoPD-grafted SiO_2_ composite (f). These spectral characteristics elucidate the FTIR graph, as the absorption bands directly reflect the vibrational modes of chemical bonds within the CT-cross-linked PoPD composite. The similarities in peak positions with CT validate the preservation of the basic CT framework, while variations in peak intensities indicate successful cross-linking and interaction with PoPD. Therefore, the FTIR spectra not only exhibit characteristic peaks but also unveil the underlying chemical interactions responsible for the graph’s formation.

#### 3.1.2. XRD Studies

**Figure 2:**
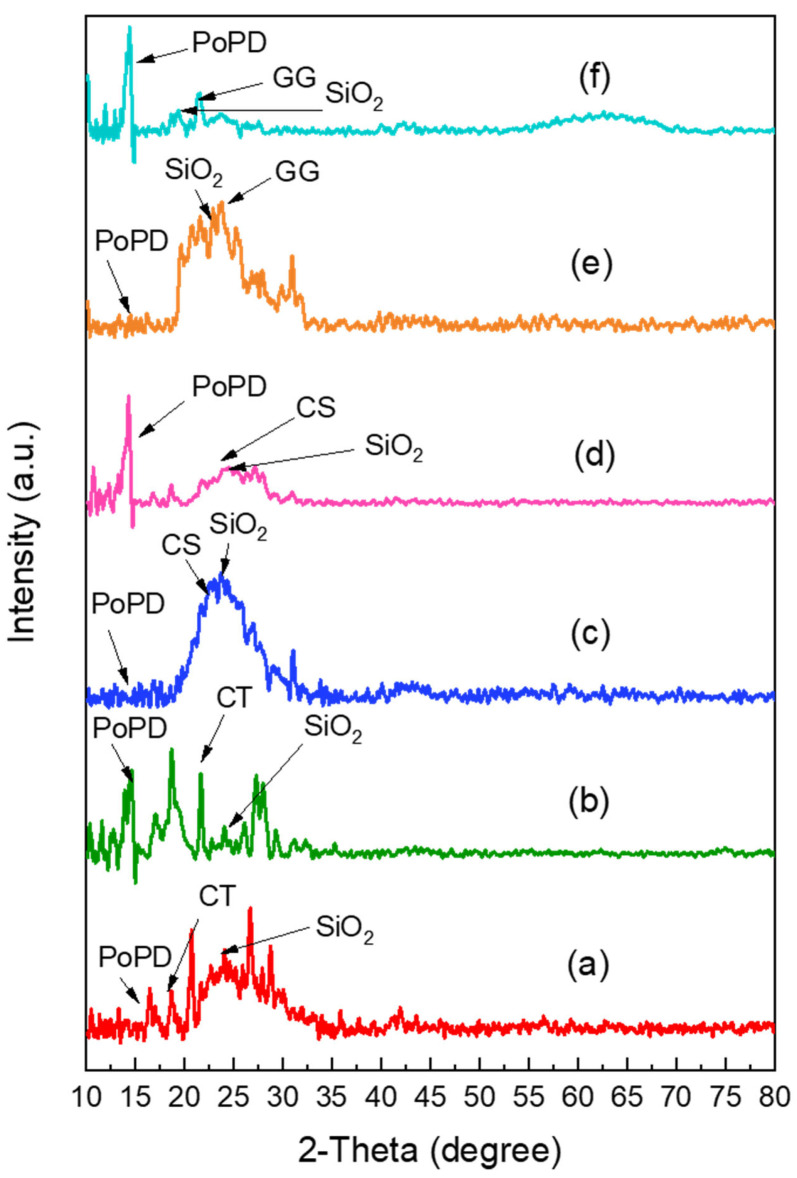
X-ray diffraction of CT-cross-linked PoPD composite (**a**), CT-PoPD-grafted SiO_2_ composite (**b**), CS-cross-linked PoPD composite (**c**), CS-PoPD-grafted SiO_2_ composite (**d**), GG-cross-linked PoPD composite (**e**), and GG-PoPD-grafted SiO_2_ composite (**f**).

XRD patterns of CT-cross-linked PoPD composite (a), CT-PoPD-grafted SiO_2_ composite (b), CS-cross-linked PoPD composite (c), CS-PoPD-grafted SiO_2_ composite (d), GG-cross-linked PoPD composite (e), and GG-PoPD-grafted SiO_2_ composite (f) are depicted in Figure 2. The XRD patterns were collected within the 2θ range of 10–80. In Figure 2a–f, the XRD analysis reveals two broad band peaks centered at 16.47 and 17.38 between 15 and 36, indicating partial crystallinity of PoPD [45]. Figure 2a,b demonstrates strong crystalline peaks at 2θ = 9.1° and 19.1° for CT [39]. A broader diffraction peak at 2θ = 21.5° is observed in Figure 2c,d for CS, indicating its characteristic peak. The XRD pattern of GG displays a broad hump at 2θ = 23°, suggesting its amorphous nature, as shown in Figure 2e,f. Figure 2b,d,f presents the XRD patterns for the SiO_2_ nanoparticles, showing a distinct peak at approximately 2θ ≈ 21°–23°, confirming the presence of the polymer-grafted SiO_2_ composite [46]. In this study, SiO_2_ influenced the crystallization of cross-linked PoPD composite polymers as a nucleating agent [47] and affected the formation of hydrogen bonds in polymers through its interaction with amino and hydroxyl groups. The change in hydrogen bonds led to a modification in the crystallinity of the polymers. The addition of SiO_2_ resulted in less intense and sharper diffraction peaks of polymer-grafted SiO_2_ nanocomposites compared to those of the cross-linked PoPD composite, indicating an enhancement in the crystallinity of the polymer-grafted SiO_2_ nanocomposites. Conversely, some peaks nearly disappeared. The improved crystallinity of the polymer-grafted SiO_2_ nanocomposites is likely due to the removal of amorphous regions from the cross-linked PoPD composite [48]. The results suggest that the increased concentration of crystalline SiO_2_ within the polymer matrix is responsible for the enhanced crystallinity observed in polymer-grafted SiO_2_ nanocomposites.

#### 3.1.3. SEM and EDX Studies

**Figure 3:**
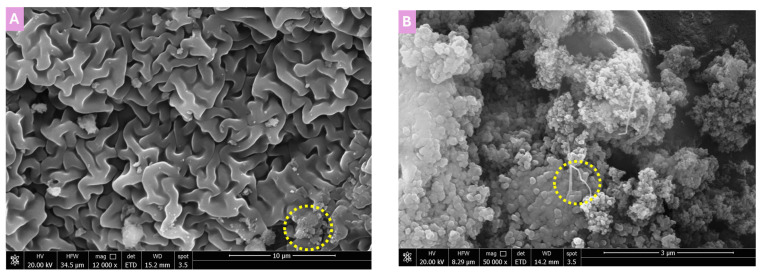

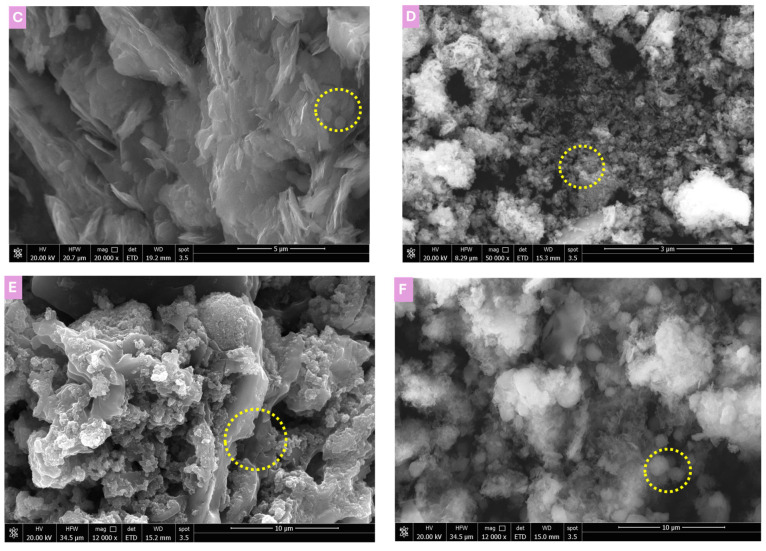
SEM images of CT-cross-linked PoPD composite (**A**), CT-PoPD-grafted SiO_2_ composite (**B**), CS-cross-linked PoPD composite (**C**), CS-PoPD-grafted SiO_2_ composite (**D**), GG-cross-linked PoPD composite (**E**), and GG-PoPD-grafted SiO_2_ composite (**F**).

**Figure 4:**
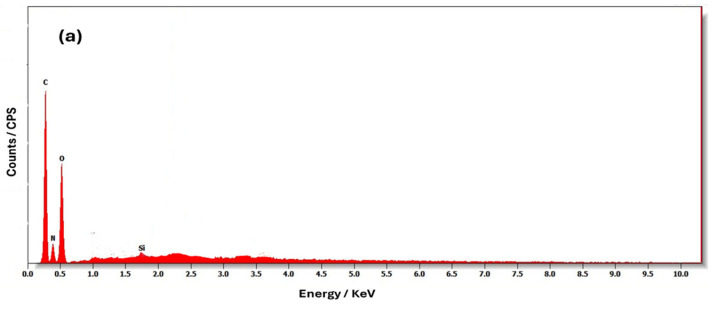

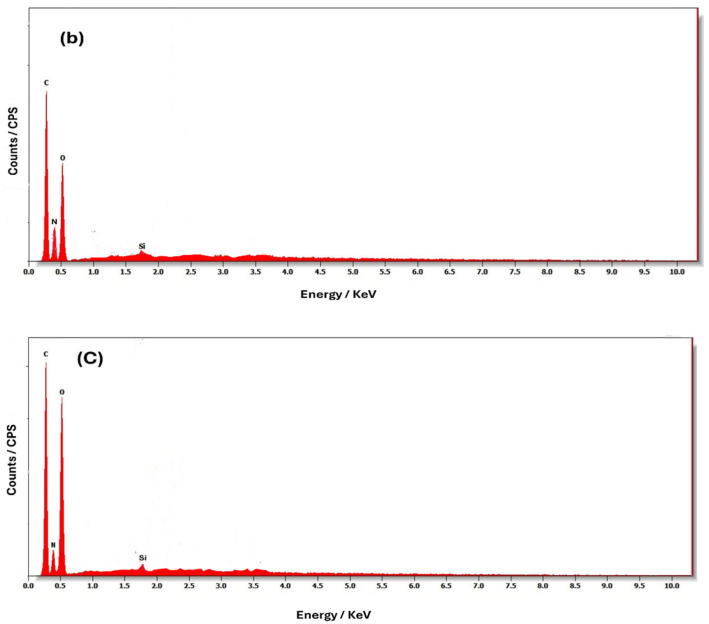
EDX of CT-PoPD-grafted SiO_2_ composite (**a**), CS-PoPD-grafted SiO_2_ composite (**b**), GG-PoPD-grafted SiO_2_ composite (**c**).

The microstructure of the CT-cross-linked PoPD composite (A), CT-PoPD-grafted SiO_2_ composite (B), CS-cross-linked PoPD composite (C), CS-PoPD-grafted SiO_2_ composite (D), GG-cross-linked PoPD composite (E), and GG-PoPD-grafted SiO_2_ composite (F) is depicted in Figure 3. Figure 3A shows wrinkles and a well-connected three-dimensional porous structure. Macropores are randomly distributed throughout the CT-cross-linked PoPD composite. Upon the integration of SiO_2_, the CT-PoPD-grafted SiO_2_ composite (B) displays an extremely porous and interlinked structure. Concurrently, the microstructure of the material transitions into a lamellar configuration with the addition of SiO_2_ (Figure 3B). The images in Figure 3 are magnified representations of different samples, demonstrating that the incorporation of SiO_2_ enhances surface roughness. As shown in Figure 3C, the CS-cross-linked PoPD composite exhibits a lamellar structure with a few folds. After the reaction with SiO_2_ (Figure 3D), the CS-PoPD-grafted SiO_2_ composite shows agglomerates and several relatively large porous particles, leading to a decrease in surface flatness. The SEM image of the GG-cross-linked PoPD composite (Figure 3E) shows a sponge-like morphology, indicating successful polymerization of the polymers. The structured surface of the GG-cross-linked PoPD composite undergoes changes after the grafting of SiO_2_ onto the polymeric backbone, resulting in a heterogeneous, rigid, and dispersed morphology conducive to dye adsorption, as depicted in Figure 3F. The image of the GG-PoPD-grafted SiO_2_ composite illustrates the porous nature of the surface, crucial for optimal adsorption performance. Figure 4 presents the EDX analysis for the CT-PoPD-grafted SiO_2_ composite (a), CS-PoPD-grafted SiO_2_ composite (b), and GG-PoPD-grafted SiO_2_ composite (c), confirming the presence of specific elements in the samples. The detected peaks for O, N, C, and Si indicate the presence of SiO_2_, PoPD, and natural polymers within the three composites.

#### 3.1.4. XPS Studies

**Figure 5:**
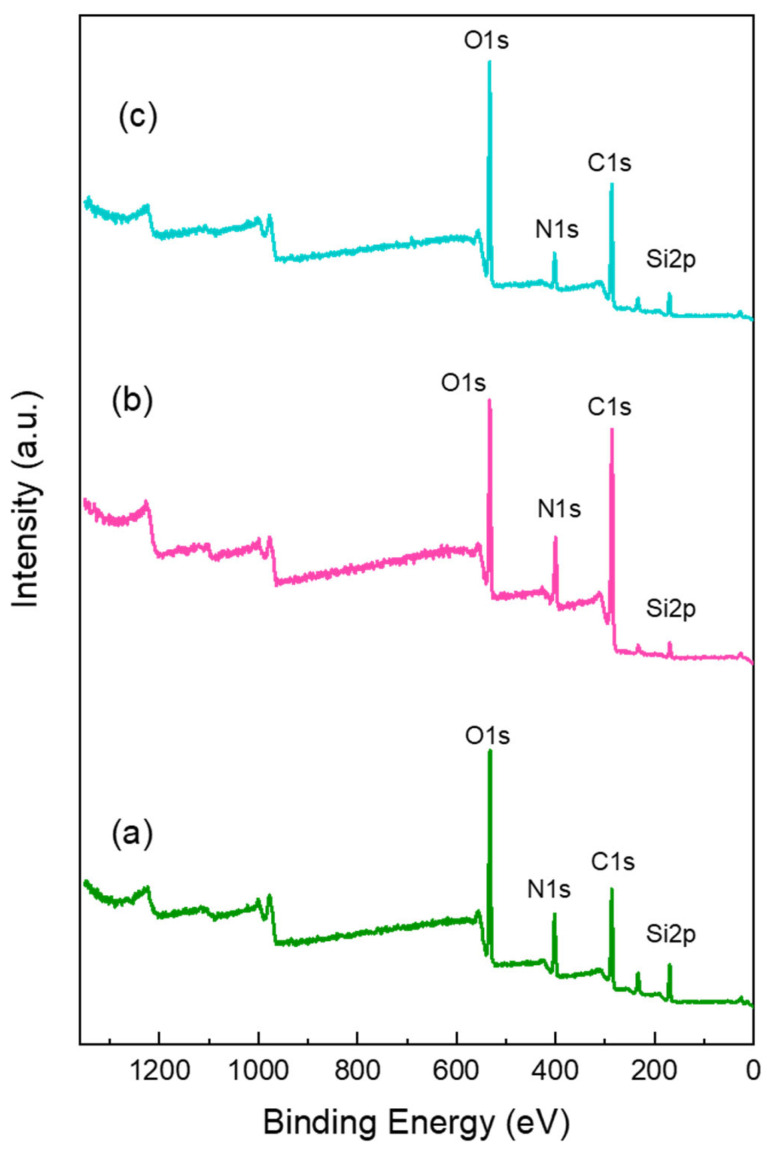
XPS of CT-PoPD-grafted SiO_2_ composite (**a**), CS-PoPD-grafted SiO_2_ composite (**b**), GG-PoPD-grafted SiO_2_ composite (**c**).

XPS was used to analyze the surface elemental composition of the CT-PoPD-grafted SiO_2_ composite (a), CS-PoPD-grafted SiO_2_ composite (b), and GG-PoPD-grafted SiO_2_ composite (c). The XPS survey spectra are presented in Figure 5. The XPS spectra of the three composites exhibited prominent peaks with binding energies at 286 eV (C), 400 eV (N), 533 eV (O), and 170 eV (Si). These characteristic peaks closely resembled the XPS data of CT reported in earlier studies [49], CS [41], GG [43], PoPD [50], and SiO_2_ [51], indicating the presence of three natural polymer-PoPD-grafted SiO_2_ composites.

### 3.2. Adsorption Studies 

**Figure 6:**
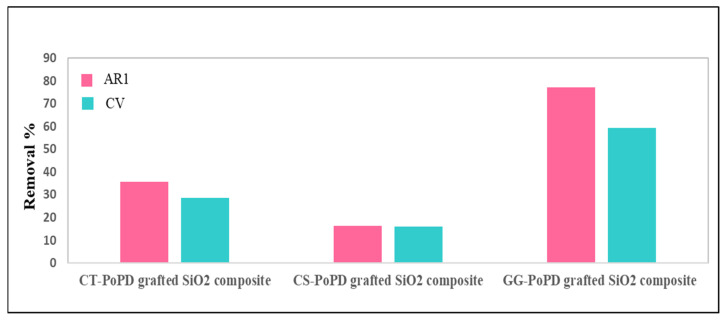
Efficiency comparison of CT-PoPD-grafted SiO_2_ composite, CS-PoPD-grafted SiO_2_ composite, and GG-PoPD-grafted SiO_2_ composite, in removing AR1 and CV dyes from an aqueous phase.

**Figure 7:**
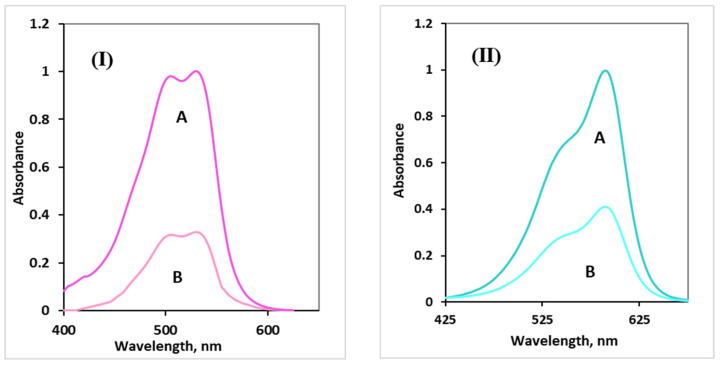
The electronic spectra of (**I**) (A) AR1 dye (20 ppm) in the sample solution without GG-PoPD-grafted SiO_2_ composite, (B) after shaking in solution with GG-PoPD-grafted SiO_2_ composite (15 mg) and (**II**) (A) CV dye (5 ppm) in the sample solution without GG-PoPD-grafted SiO_2_ composite, (B) after shaking in solution with GG-PoPD-grafted SiO_2_ composite (15 mg).

Figure 6 shows the percentages of AR1 and CV dyes removed after testing the efficacy of the CT-PoPD-grafted SiO_2_ composite, CS-PoPD-grafted SiO_2_ composite, and GG-PoPD-grafted SiO_2_ composite in eliminating these dyes from an aqueous solution. The GG-PoPD-grafted SiO_2_ composite demonstrates superior efficiency (Figure 6) compared to other samples. The enhancement of functional groups on the GG-PoPD-grafted SiO_2_ composite appears effective due to the presence of three rings in the GG structure, which enhances adsorption capacities. The delocalization of π-electrons facilitates chelation, leading to the removal of various environmental contaminants, including dyes. The significant surface area, robust removal capability, and interactions resulting from surface electrostatic charges among PoPD, SiO_2_, and dyes [52,53,54,55,56] further amplify this effect. Based on these findings, the GG-PoPD-grafted SiO_2_ composite was chosen for subsequent experiments investigating various parameters. The electronic spectra of AR1 and CV dyes in aqueous solutions exhibit absorption peaks at 530 nm and 590 nm, respectively (Figure 7I and II). These peaks’ intensity notably decreases upon interaction with the GG-PoPD-grafted SiO_2_ composite, indicating the composite’s effectiveness in removing the dyes from water. This study aimed to assess the GG-PoPD-grafted SiO_2_ composite’s efficiency in removing AR1 and CV dyes from water-based solutions using an adsorbent. The effects of temperature, adsorbent dosage, contact time, and pH on removal efficiency were examined. Subsequently, adsorption kinetics were analyzed to explore the relationship between the adsorption behavior of AR1 and CV dyes and the composition of the adsorbent. This study investigates the efficacy of three distinct natural polymers in dye removal by combining them with PoPD grafted with SiO_2_. This method produces adsorbents with high mechanical strength, a large specific surface area of the NCs, and substantial adsorption capacity for efficient dye removal from wastewater.

### 3.3. Optimization of Various Parameters 

#### 3.3.1. Effect of PH 

**Scheme 1:**
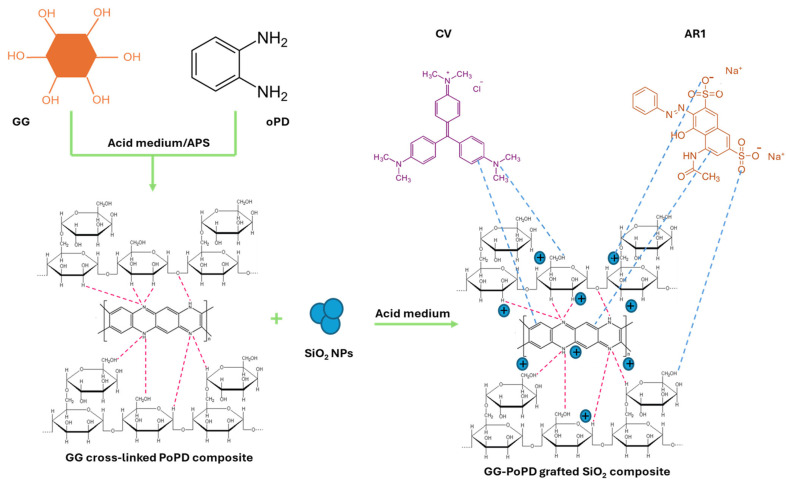
Illustration of the proposed mechanism for manufacturing GG-PoPD-grafted SiO_2_ composite.

**Figure 8:**
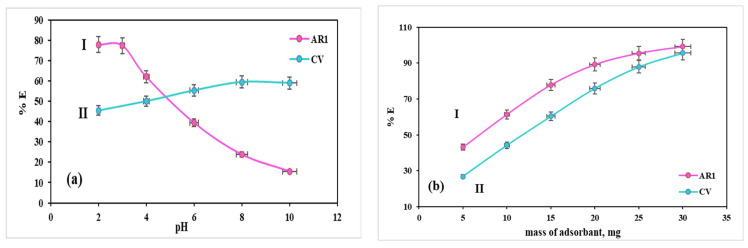

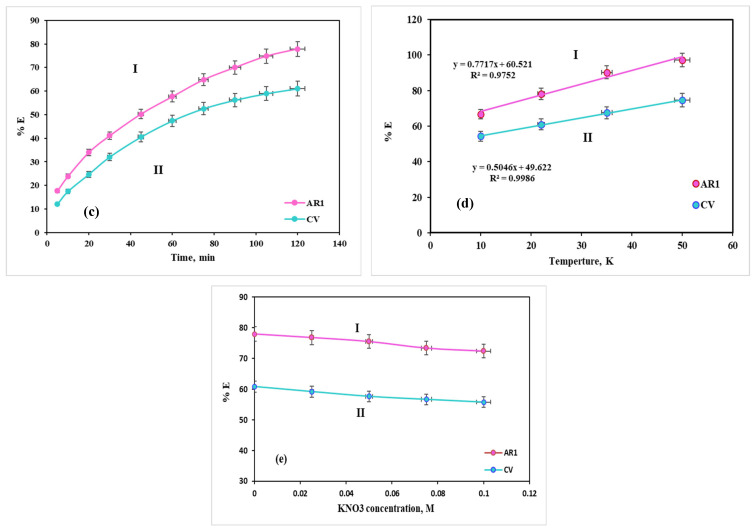
Optimization of parameters for the adsorbent GG-PoPD-grafted SiO_2_ composite of dyes AR1 (I) and CV (II) from the solution samples. Optimization of effect of pH (the solution samples upon (0.015 ± 0.0003 g) composite (**a**), Amount of material (**b**), contact time (**c**), Temperature (**d**) and Influence of concentration of KNO_3_ (**e**). (contact time 120 min and at 22 °C temperature).

By adjusting the pH of the AR1 and CV dye solutions from acidic to alkaline conditions, we investigated the impact of pH on the optimal conditions for dye removal. The pH levels were modified by adding solutions of HCl or NaOH, resulting in acidic or alkaline environments. The results, illustrated in Figure 8a, indicate that the removal percentage of AR1 dye is significantly higher at pH levels of 2–3, with a notable decrease as pH increases. Conversely, for CV dye, increasing the pH of the solution enhances the absorption percentage onto GG-PoPD-grafted SiO_2_ composite SP up to pH 8, after which a decline is observed, as shown in Figure 8a. This suggests a potential interpretation of the results: The GG-PoPD-grafted SiO_2_ composite facilitates the adsorption of anionic dye molecules in acidic and slightly alkaline solutions due to increased surface protonation resulting from higher H^+^ ion concentration. An increase in the negative charge density of the adsorbent surface under alkaline conditions creates repulsive forces that hinder the removal of anionic dyes. Although direct evidence of surface charge (e.g., zeta potential measurements) was not within the scope of this study, the observed adsorption behavior at different pH values indirectly supports this explanation. Future studies will aim to include zeta potential characterization to further validate this mechanism. The reduced dye removal rate in very alkaline solutions is attributed to the composite surface attracting hydroxyl ions, which neutralize surface charges and restrict dye adsorption [57]. Various interactions may contribute to this phenomenon: (1) Hydrophilic groups on the composite surface can form hydrogen bonds with functional groups on the molecules, such as –OH from GG, -NH from PoPD, –N from CV dye, and –O and –NH from AR1. (2) The aromatic components of the dyes can engage in π–π interactions with the benzene rings of PoPD. (3) In acidic conditions, anionic dyes can establish electrostatic attractions with the positively charged surfaces of the composites, through the –NH_2_ of PoPD and the –OH of GG. Notably, a significant interaction is observed between the oxygen atoms of the dye molecules and SiO_2_ nanoparticles [58], as illustrated in Scheme 1. At low pH levels, the GG-PoPD-grafted SiO_2_ composite adsorbents exhibit a positive surface charge, while their surface negative charge density increases with increasing pH levels. Owing to repulsive interactions, the anionic dye AR1 cannot be fully removed from a basic solution using the GG-PoPD-grafted SiO_2_ composite adsorbent. AR1 dye is best adsorbed at higher pH values due to the strong electrostatic interaction between the positively charged GG-PoPD-grafted SiO_2_ composite and the negatively charged dye molecules. In acidic environments, the cationic dye CV shows lower absorption as its molecules are repelled by the positively charged GG-PoPD-grafted SiO_2_ composite. Conversely, the cationic CV dye exhibits improved absorption at higher pH values due to favorable interactions between the positively charged dye molecules and the negatively charged GG-PoPD-grafted SiO_2_ composite, followed by reduced absorption at subsequent pH levels. Our findings indicate that a pH of 2, adjusted using 0.1 M HCl, is optimal for AR1 dye adsorption, while a pH of 8 is ideal for CV dye adsorption. This observation highlights that adsorption efficiency is primarily influenced by surface charge modulation of the adsorbent, electrostatic interactions, hydrogen bonding, and π–π interactions between the dye molecules and the composite.

#### 3.3.2. Effect of Adsorbent Dosage 

Figure 8b presents the results of a study investigating the effect of the mass of the GG-PoPD-grafted SiO_2_ composite on the adsorption efficiency of AR1 and CV dyes at concentrations of 20 mg/L and 5 mg/L, respectively. The findings revealed a significant enhancement in the removal ratio of AR1 dye from 43.1% to 99.1% when increasing the GG-PoPD-grafted SiO_2_ composite dose from 5 mg to 30 mg. Similarly, for CV dye, the percentage increased from 26.8% to 95.6% with the dose escalation from 5 mg to 30 mg. This increase in percentage indicates an increase in active sites available for adsorption, facilitating greater interaction between dye molecules and the composite surface. The study utilized 15 mg of GG-PoPD-grafted SiO_2_ composite to eliminate AR1 and CV dyes, achieving removal efficiencies of 77.8% and 60.4%, respectively. This approach enabled the assessment of various additional factors influencing the adsorption process.

#### 3.3.3. Contact Time 

Dye adsorption experiments heavily depend on contact time as a crucial variable. In this study, experiments were carried out with contact times ranging from 0 to 120 min. Figure 8c depicts the effectiveness of the GG-PoPD-grafted SiO_2_ composite adsorbent in removing dye. Both dyes showed increasing removal percentages over time, with a significant amount of adsorption occurring within the initial 75 min. This indicates rapid transfer of dye molecules from the solution to the composite surface during the early stages of the process. The swift initial adsorption is attributed to the immediate availability of active surface sites. Subsequently, the adsorption rate slowed considerably, likely due to the gradual saturation of these sites, suggesting the system was approaching equilibrium. However, full equilibrium was not reached within the 120 min timeframe, as a gradual increase in dye removal persisted.

#### 3.3.4. Temperature 

Adsorption experiments heavily depend on temperature. In this study, a temperature range of 280–318 K was utilized to investigate the adsorption process, as shown in Figure 8d. The removal efficiency peaked at 315 K. The GG-PoPD-grafted SiO_2_ composite demonstrates effective removal efficiencies for AR1 and CV dyes at elevated temperatures. The adsorption process is endothermic, indicating that higher temperatures facilitate molecular mobility and enhance interactions between dye molecules and the composite surface, thereby increasing adsorption.

#### 3.3.5. Effect of Ionic Strength 

The interaction between GG-PoPD-grafted SiO_2_ composites and AR1 and CV dyes is influenced by the ionic strength. It is essential to investigate how external electrolytes, like KNO_3_, impact the interaction between dyes and composite materials since salts are common in effluents from the textile industry. This study explores the influence of varying concentrations of KNO_3_ solution on the absorption of CV and AR1 dyes by GG-PoPD-grafted SiO_2_ composites (Figure 8e). The efficiency of dye removal was slightly reduced at higher KNO_3_ concentrations based on the provided data. The increased charge density on the surface of the adsorbed solid phase hinders dye adhesion, especially in the presence of cations such as K^+^ [15,16]. This highlights the importance of considering ionic competition and alterations in surface charge density, which diminish electrostatic attraction and subsequently lower adsorption efficiency at higher ionic strengths.

### 3.4. Kinetic Study

**Figure 9:**
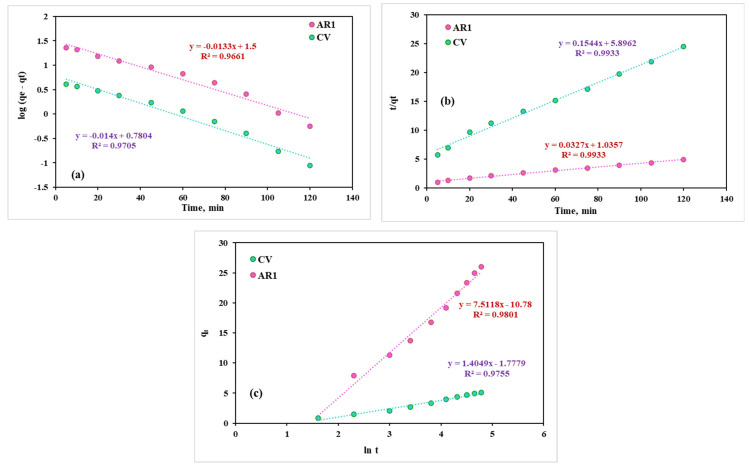
Kinetic models for the adsorption removal of AR1 and CV by GG-PoPD-grafted SiO_2_ composite: (**a**) Lagergren plot, (**b**) Pseudo-second-order plot, (**c**) Elovich model plot.

Graphing log(q_e_ − q_t_) and t/q_t_ vs. t was utilized to evaluate the adequacy of modeling the entire adsorption process, as depicted in Figure 9a,b. Figure 9c shows the Elovich model’s plot of q_t_ versus ln t, while Table 1 presents the regression coefficients (R^2^) of the resulting linear equations, along with the α and β parameters of the composite. Both the PFO and Elovich models fail to accurately capture the kinetics of the adsorption process, evident from the R^2^ values. The positive regression coefficient shown in Figure 9b indicates that the PSO kinetic model serves as a valuable tool for analyzing the adsorption process. Table 1 displays the estimated values of q_e_, k_1_, and k_2_ derived from the intercepts and slopes of the two equations. The quantities of CV and AR1 adsorbed per unit mass of the GG-PoPD-grafted SiO_2_ composite at equilibrium (q_e,calc_) exhibit significant agreement with the experimentally determined values of the PSO model (q_e,exp_), as indicated in Table 1. This consensus confirms the suitability of the PSO model for elucidating the adsorption procedure [15,59].

### 3.5. Thermodynamic Parameters of AR1 and CV Dye Adsorption on the GG-PoPD-Grafted SiO_2_ Composite 

**Figure 10:**
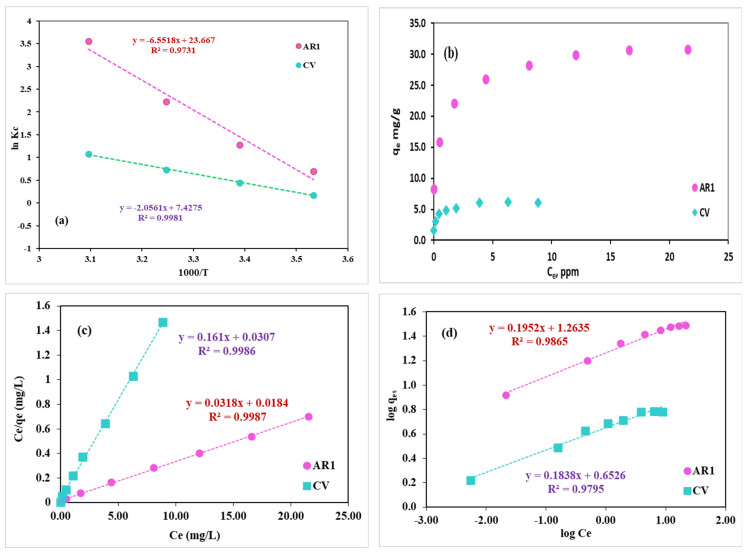
Curves of ln Kc against 1000/T for uptake AR1 and CV dye from aquatic solution by GG-PoPD-grafted SiO_2_ composite (**a**), Plot of AR1 and CV dyes retained (q_e_) onto the GG-PoPD-grafted SiO_2_ composite solid phase (mg/g) against equilibrium concentration (C_e_), at 22 °C (**b**), Langmuir sorption isotherms of AR1 and CV dyes uptake onto the GG-PoPD-grafted SiO_2_ composite at 22 °C (**c**), Plot of log C_ads_ vs. log C_e_ of AR1 and CV dyes retention onto GG-PoPD-grafted SiO_2_ composite at 22 °C. (Freundlich isotherms) (**d**).

The impact of temperature on the adsorption of the two dyes (AR1 and CV) was evaluated in the temperature range of 285–318 K. The relevant thermodynamic parameters are presented in Table 2. To investigate the removal of AR1 and CV dyes with temperature variations, ΔH, ΔG, and ΔS were determined by analyzing the intercept and slope of the ln Kc versus 1000/T graph, respectively. Figure 10a illustrates a linear curve, indicating that the adsorption thermodynamic parameters (ΔH and ΔS) can be directly derived from the slope and intercept of the line. This confirms the method’s reliability and suggests that the adsorption behavior is temperature-dependent. The results in Table 2 suggest an endothermic adsorption process, as evidenced by the increase in Kc with temperature. The positive ΔH value validates the endothermic dye adsorption of CV and AR1 compounds on the GG-PoPD-grafted SiO_2_ composite. The positive ΔS value indicates an increase in randomness at the solid–solution interface for both CV and AR1 dyes. The adsorption process is favorable and spontaneous when the calculated Gibbs free energy (ΔG) values are negative.

### 3.6. Adsorption Isotherms for AR1 and CV Dyes Uptake by the GG-PoPD-Grafted SiO_2_ Composite

The interaction between AR1 and CV dyes on the GG-PoPD-grafted SiO_2_ composite sorbent layer was elucidated through sorption isotherms. Equilibrium studies are essential for determining key surface properties of the sorbent and the maximum adsorption capacity of the composite for AR1 and CV dyes, depending on the specific adsorption mechanism employed. A comprehensive analysis was conducted on the retention patterns in aqueous solutions using the selected sorbent under optimal conditions, ranging from 5 to 40 mg/L for AR1 and 1 to 12.5 mg/L for CV dyes. At equilibrium concentrations of AR1 and CV dyes, the amount of dye retained on the composite is depicted in Figure 10b. A linear relationship was observed between the number of dyes incorporated in the composite and the amount retained in the solution at moderate sample concentrations. This linear correlation indicates that, within the investigated range, the quantity of available active sites on the composite is directly proportional to the equilibrium concentration of the dyes, resulting in incremental adsorption until reaching saturation. The composite exhibited an adsorption capacity of 6.13 ± 0.09 mg/g for CV and 30.75 ± 0.12 mg/g for AR1.

The Langmuir isotherm equation, used to assess the adsorption of AR1 and CV dyes on a composite sorbent, is expressed by the following linear form [60]:

(3) $$\frac{C_{e}}{q_{e}}=\frac{1}{q_{m}k_{L}}+\frac{C_{e}}{q_{m}}$$

C_e_ represents the equilibrium concentration of the dyes (in milligrams per liter) in the solution under investigation, while q_e_ denotes the quantity of solute adsorbed per mass of adsorbent at equilibrium (in milligrams per gram). The parameters q_m_ and K_L_ signify the maximum solute capacity for adsorption per unit mass of adsorbent necessary for monolayer surface coverage and the temperature-independent binding energy of solute adsorption, respectively, in the Langmuir model. Figure 10c illustrates a linear correlation between Ce/q_e_ and Ce for all AR1 and CV dye concentrations on the composite. This linear trend confirms that adsorption takes place as a monolayer process on a uniform surface with identical active binding sites, aligning with the Langmuir model’s assumptions. The R^2^ values for AR1 dye were 0.9987 and for CV dye were 0.9986, indicating that the adsorption of these dyes onto the composite surface is uniform and adheres closely to the Langmuir model. Table 3 presents the values of q_m_ and KL derived from the slope and intercept of the linear graph. The characteristic feature of this model, the dimensionless separation factor, or R_L_, is expressed as follows:

(4) $$R_{L}=\frac{1}{1+\;K_{L}C_{o}}$$

In the case of AR1 and CV dyes, the R_L_ values for the adsorbent are 0.079 and 0.208, respectively, indicating excellent monolayer adsorption, as stated in a previous study [61].

Using the Freundlich model, which is expressed in the following linear form [62], we evaluated the retention properties of AR1 and CV dyes from aqueous solutions on the utilized sorbents.

(5) $$\log q_{e}=logK_{F}+\left(1/n\right)\log C_{e}$$

To determine the maximum solute sorption capacity (mg/g), the Freundlich isotherm parameters K_F_ and 1/n can be utilized. q_e_ represents the retained AR1 and CV dye concentration on the GG-PoPD-grafted SiO_2_ composite per unit mass (mg/g) at equilibrium, while C_e_ denotes the dye concentration in the aqueous mixture (mg/L). The Freundlich parameters K_F_ and 1/n, obtained from the intercept and slope in Figure 10d, are detailed in Table 3. Both AR1 and CV dyes exhibited 1/n values below one, specifically 0.195 and 0.184, respectively, indicating effective sorption of these dyes by the GG-PoPD-grafted SiO_2_ composite surfaces. The slope in Figure 10d corresponds to the 1/n value, confirming the heterogeneous nature of the adsorption sites as it is less than 1, while the intercept provides K_F_, reflecting the composite’s adsorption capacity. This trend suggests that adsorption intensity diminishes with increasing solute concentration, a characteristic feature of the Freundlich model. The correlation coefficients (R^2^) of the Freundlich model for AR1 and CV dyes, as shown in Table 3, do not reach sufficiently high values, suggesting that the Langmuir model offers a more precise depiction of the adsorption process.

### 3.7. Comparison with Other Adsorbents

Table 4 compares the GG-PoPD-grafted SiO_2_ composite to other adsorbents reported in the literature in terms of pH levels, temporal characteristics, and adsorption capacities. This study demonstrates that the GG-PoPD-grafted SiO_2_ composite is highly effective in extracting AR1 and CV dyes from solutions, serving as an alternative to traditional solid adsorbents. The composite material exhibits exceptional adsorption capabilities due to its innovative design, incorporating various components with significant adsorbent capacity. This design enhances the overall adsorbent capacity and increases the number of active sites, positioning the GG-PoPD-grafted SiO_2_ composite as a noteworthy option in adsorption techniques. Moreover, the adsorption capacity of the GG-PoPD-grafted SiO_2_ composite surpasses that of many commercial or previously documented adsorbents, as indicated in Table 4. This highlights its superior performance in dye removal efficiency. Additionally, the utilization of cost-effective, biodegradable, and readily available precursors such as guar gum and silica enhances the economic viability of the synthesized composite, rendering it a promising candidate for practical applications in wastewater treatment.

### 3.8. Application of the GG-PoPD-Grafted SiO_2_ Composite for the Removal of Dyes from Real Water Samples

**Figure 11:**
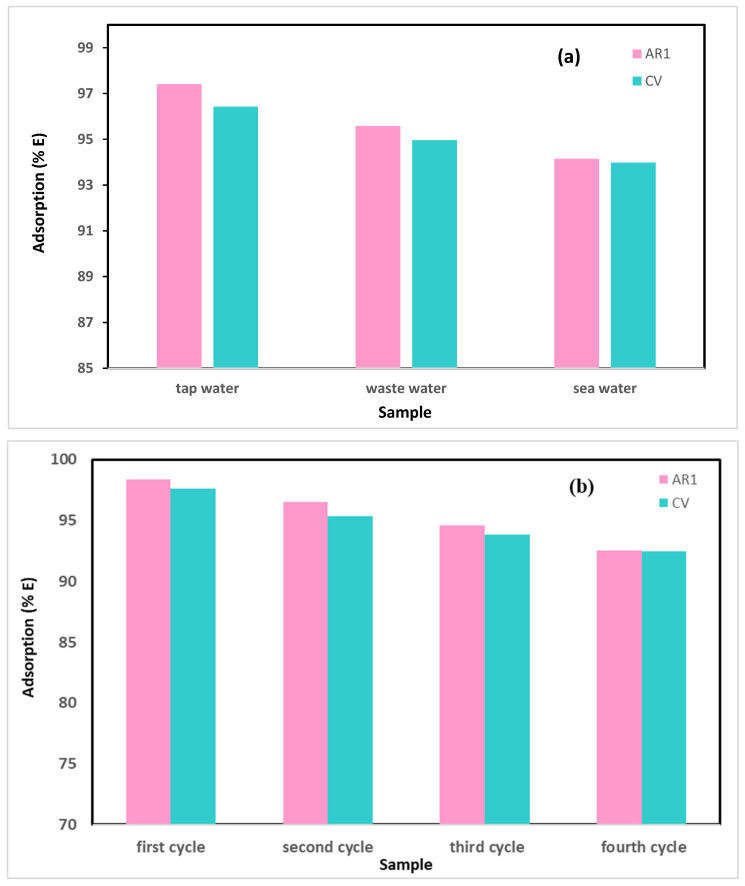
The efficiency of AR1 and CV dyes removed by GG-PoPD-grafted SiO_2_ composite from various real three samples (**a**), removal performance of GG-PoPD-grafted SiO_2_ composite towards AR1 and CV dyes over four cycles (**b**).

Adsorption has recently gained popularity due to its cost-effectiveness compared to other wastewater treatment methods. The efficiency of the adsorbent material in removing actual pollutants from is crucial for practical applications. The complex structure of real water can pose challenges to the adsorption process when dyes interact with the adsorbent. Various adsorption tests were conducted using authentic water samples, encompassing freshwater, saltwater, and wastewater, to evaluate the performance of the adsorbent material in practical scenarios. The utilization of real water samples, including freshwater, saltwater, and wastewater, allows for the consideration of diverse salts, ions, and contaminants. These naturally occurring components may compete with dye molecules for adsorption sites, impacting the removal efficiency. Consequently, the outcomes offer a pragmatic assessment of the adsorbent’s efficacy under intricate water matrices representative of actual environmental conditions. Section 2.6 delineates the methodologies employed for sample analysis. The analysis of the three samples indicated the presence of AR1 and CV dyes above the detection limit of UV–vis spectroscopy. Figure 11a and Table 5 elucidate the experimental setup, while the percentage recoveries are presented. The slightly superior removal efficiency of AR1 in comparison to CV can be attributed to its smaller molecular size and enhanced accessibility to active binding sites in AR1, facilitating a stronger interaction with the functional groups of the GG-PoPD-grafted SiO_2_ composite. This evaluation gauges the efficacy of the adsorbent material using real water samples in a practical context.

### 3.9. Recycling Tests

An important economic factor is the potential for the compound to be reused multiple times in adsorption processes. To assess the reusability of the GG-PoPD-grafted SiO_2_ composite, four cycles of adsorption and desorption were conducted. Despite a slight decrease in desorption efficiency with each cycle, Figure 11b shows that even after four cycles, a significant level of AR1 and CV removal efficiency was maintained. This discovery enhances the economic viability of the GG-PoPD-grafted SiO_2_ composite by demonstrating its sustained effectiveness across multiple adsorption–desorption cycles.

## 4. Conclusions

To effectively eliminate the dyes AR1 and CV, this study outlines the development of CT-, CS-, and GG-PoPD-grafted SiO_2_ composites. The formation of the composites was confirmed through FTIR, XRD, FESEM, and XPS analyses. FESEM analysis revealed that the GG-PoPD-grafted SiO_2_ composite exhibits a porous structure with a larger surface area compared to the CT-PoPD- and CS-PoPD-grafted SiO_2_ composites. Adsorption peaked at pH = 2 for AR1 and at pH = 8 for CV. Results of the kinetic studies suggested that the adsorption rate is affected by the amount of dye adsorbed, indicating alignment with a PSO kinetic model. The GG-PoPD-grafted SiO_2_ composite exhibited maximum absorption capacities of 25.98 mg/g for AR1 and 5.089 mg/g for CV. Thermodynamic analyses demonstrated that the adsorption of AR1 and CV is spontaneous, favorable, and endothermic. The composite can be effectively regenerated while maintaining its adsorption capabilities, positioning the GG-PoPD-grafted SiO_2_ composite as a viable option for wastewater treatment applications.

## Figures and Tables

**Table 1:** Kinetics model constants and determination coefficients for AR1 and CV adsorption by GG-PoPD-grafted SiO_2_ composite sorbents at 22 °C.

The Pseudo-First-Order Kinetic (Lagregen) Model				
	q_e, exp_ (mg/g)	q_e, calc_ (mg/g)	k_1_	R^2^
AR1	25.98	31.62	0.031	0.966
CV	5.089	6.029	0.032	0.971
**The Pseudo-Second-Order Kinetic Model**				
	**q_e, exp_ (mg/g)**	**q_e, calc_ (mg/g)**	**k_2_**	**R^2^**
AR1	25.98	30.58	1.03 × 10^−3^	0.993
CV	5.089	6.47	4.0 × 10^−3^	0.993
**Elovich Kinetic Model**				
	**α, (g/mg min)**	**β, (mg/g min)**		**R^2^**
AR1	0.032	7.51		0.98
CV	0.181	1.405		0.976

**Table 2:** The parameters of thermodynamic for AR1 and CV dyes through (285–318 K).

Equations to Calculate the Thermodynamic Parameters	Dye	ΔH (kJ/mol)	ΔS (J/K mol)	ΔG (kJ/mol)
$\ln Kc=\frac{-\Delta H}{RT}+\frac{\Delta S}{R}$ ΔG = ΔH − TΔS ΔG = RT lnK_c_ $k_{c}=\frac{C_{a}}{C_{e}}$	AR1	54.47	196.7	−3.556
	CV	17.09	61.75	−1.122

ΔH = enthalpy change (J mol^−1^), ΔS = entropy change (J mol^−1^), ΔG = Gibbs free energy (J mol^−1^), C_e_ = concentration at equilibrium for dyes in aqueous solution (mg L^−1^), C_a_ is the concentration of dyes uptake onto the composite per liter at equilibrium (mg/L), q_e_ is quantity of adsorbed dyes per unit weight of GG-PoPD-grafted SiO_2_ composite at equilibrium (mg/g), R = universal gas constant (8.314 J K^−1^ mol^−1^), T = temperature (K), K_C_ = equilibrium constant.

**Table 3:** The parameter of Langmuir and Freundlich isotherm models for the retention of AR1 and CV dyes on GG-PoPD-grafted SiO_2_ composite solid phase at 22 °C.

Models of Sorption Isotherms		AR1 Dye	CV Dye
Langmuir	q_m_, mg/g	31.45	6.21
	K_L_, L/g	0.578	0.191
	R_L_	0.079	0.208
	R^2^	0.9987	0.9986
Freundlich	K_F_, mg/g	18.32	4.89
	1/n	0.195	0.184
	R^2^	0.9865	0.9795

**Table 4:** Removal capacity, time, and PH for adsorption removal of AR! and CV dyes using various adsorbents.

Dye	Adsorbents	Shaking Time (min)	pH	q_e_ calc (mg/g)	Reference
AR1	Halloysite nanoclay	60	2	13.89	[63]
	Sunflower seed shell	100	2	6.81	[64]
	Polypyrrole/Mn0.8Zn0.2Fe_2_O_4_/Graphite Oxide ternary composites	60	2	2.81	[55]
	GG-PoPD-grafted SiO_2_ composite	75	2	30.58	This Work
CV	a marine diatom alga, *Skeletonema costatum*	120	3	6.41	[65]
	Modified *Bambusa tulda*	60	7	4.95	[66]
	biosorbent (white clover: *Trifolium repens*) stem powder	140	2	1.96	[67]
	GG-PoPD-grafted SiO_2_ composite	75	8	6.47	This Work

**Table 5:** Display the condition of the percentage recoveries, and the measurement of AR1 and CV in real samples.

Sample	Composite, mg		Dye, mg L^−1^		Shaken Time, min		PH		Temperature, K		Recovery, (%)	
	AR1	CV	AR1	CV	AR1	CV	AR1	CV	AR1	CV	AR1	CV
Tap water	35	35	20	5	120	120	2	8	295	295	97.41	96.42
Wastewater	35	35	20	5	120	120	2	8	295	295	95.58	94.95
Seawater	35	35	20	5	120	120	2	8	295	295	94.14	93.97

## Data Availability

The original contributions presented in this study are included in the article. Further inquiries can be directed to the corresponding author.
